# Down-regulating peroxisome proliferator-activated receptor-gamma coactivator-1beta alleviates the proinflammatory effect of rheumatoid arthritis fibroblast-like synoviocytes through inhibiting extracellular signal-regulated kinase, p38 and nuclear factor-kappaB activation

**DOI:** 10.1186/s13075-014-0472-6

**Published:** 2014-10-24

**Authors:** Jing-Jing Zhou, Jian-Da Ma, Ying-Qian Mo, Dong-Hui Zheng, Le-Feng Chen, Xiu-Ning Wei, Lie Dai

**Affiliations:** Department of Rheumatology, Sun Yat-Sen Memorial Hospital, Sun Yat-Sen University, Guangzhou, 510120 PR China

## Abstract

**Introduction:**

Rheumatoid arthritis (RA) is a chronic inflammatory disease leading to joint destruction and disability. Peroxisome proliferator-activated receptor-gamma coactivator-1beta (PGC-1β) is a transcriptional coactivator that plays important roles in regulating multiple aspects of energy metabolism and cytokine signaling pathways. PGC-1β overexpression leads to the attenuation of macrophage-mediated inflammation. In this study, we aimed to determine the expression of PGC-1β in RA synovium and fibroblast-like synoviocytes (FLS), and explore the mechanisms of PGC-1β on both the proinflammatory effects and apoptosis in RA-FLS.

**Methods:**

Synovium was obtained from 31 patients with active RA, as well as 13 osteoarthritis (OA) and 10 orthopedic arthropathies (Orth.A) as “less inflamed” disease controls. FLS were then isolated and cultured. Synovial PGC-1β expression was determined by immunohistochemistry staining, while FLS PGC-1β expression was detected by immunofluorescence staining, quantitative real-time PCR (qPCR) assay and western blot. PGC-1β was depleted by lentivirus sh-RNA, and up-regulated by pcDNA3.1- PGC-1β. The expression of proinflammatory cytokines, matrix metalloproteinases and receptor activator of nuclear factor-kappaB ligand was analyzed by qPCR, cytometric bead array and western blot. The expression of mitogen-activated protein kinases and nuclear factor-kappaB (NF-κB) was determined by qPCR and western blot. Besides, cell apoptosis was examined using flow cytometry. The interaction between PGC-1β and NF-κB was performed by dual-luciferase reporter gene assays.

**Results:**

(A) Synovial PGC-1β was over-expressed in RA patients compared with OA or Orth.A patients. (B) PGC-1β expression significantly increased in RA-FLS compared with OA-FLS. (C) PGC-1β mediated the expression of proinflammatory cytokines and apoptosis through extracellular signal-regulated kinase (ERK), p38 and NF-κB in RA-FLS. (D) PGC-1β mediated NF-κB transcription in RA-FLS, but did not affect ERK and p38.

**Conclusion:**

The results indicate that PGC-1β may play important roles in the proinflammatory effects and apoptosis of RA-FLS.

## Introduction

Rheumatoid arthritis (RA) is a common chronic inflammatory disease characterized by chronic inflammatory synovitis, leading to invasion of synovial tissue into the adjacent cartilage matrix with degradation of articular cartilage and bone [1]. RA affects 0.5% to 1.0% of the population worldwide [2]. During the last few decades, treatment options have diversified and the availability and selection of different treatments for individual patients has greatly improved. Despite these advances, a cure for RA is not yet available. Therefore, the precise etiology and underlying mechanisms of the condition require further elucidation.

Fibroblast-like synoviocytes (FLS) are recognized as a prominent joint-specific determinant of RA because of their hyperproliferative properties and hyperproduction of both proinflammatory cytokines that perpetuate inflammation and matrix metalloproteinases (MMPs) that contribute to cartilage destruction [3,4]. Briefly, proinflammatory cytokines such as TNF-α and IL-1β strongly initiate FLS, and consistently enhance and potentially stabilize the activated phenotype of FLS [5]. The activation of FLS can produce TNF-α, IL-1β and IL-6, all of which are involved in sustaining regulatory feedback loops and inducing production of MMPs, cathepsins and aggrecanases [6]. Activated FLS can also produce IL-8, which is implicated in leukocyte recruitment to diseased synovium [7]. FLS govern the differentiation of macrophages into osteoclasts through upregulation of receptor activator of nuclear factor kappa-B ligand (RANKL) [8]. This phenomenon is coordinated with cellular mitogen-activated protein kinase (MAPK) and nuclear factor-kappaB (NF-κB) signaling pathways being stimulated. These feedback loops are regarded as significant regulators of proinflammatory cytokines in RA [9,10]. As FLS are key effectors in the pathogenesis of RA, recent approaches for RA treatment have focused on the inhibition of its function and induction of cell death, including the modulation of activities of various nuclear transcriptional factors relevant to the inflammatory process [11-13].

Peroxisome proliferator-activated receptor-gamma coactivator-1 (PGC-1) is a transcriptional coactivator that plays important roles in regulating multiple aspects of energy metabolism and cytokine signaling pathways through interaction with many transcription factors [14,15]. The first member of the PGC-1 family is now termed PGC-1α, while PGC-1β is the closest homolog of PGC-1α and shares extensive sequence identity [15,16]. Emerging evidence suggests that PGC-1 plays important roles in regulating inflammation. PGC-1 can visibly suppress proinflammatory effects by inhibiting the activation of NF-κB in skeletal muscle cells [17]. PGC-1α has been demonstrated to be capable of regulating inflammation by suppressing the expression of TNF-α, IL-6, IL-8 and IL-1β mRNA levels in human skeletal muscle [18]. Overexpression of PGC-1α decreased both basal and platelet-derived growth factor-induced p38 MAPK phosphorylation [19]. More importantly, increased expression of PGC-1β resulted in the attenuation of macrophage-mediated inflammation [20]. PGC-1β is therefore a critical mediator in regulating proinflammatory cytokines. However, until now, whether PGC-1β participates in the pathophysiological development of RA has been poorly understood. This study aimed to determine the expression of PGC-1β in RA synovium and FLS, and explore the mechanisms of PGC-1β on both the proinflammatory effects and apoptosis in RA-FLS.

## Methods and materials

### Patients

Thirty-one Chinese patients with RA who fulfilled the 1987 revised criteria of the American College of Rheumatology (ACR) [21] or 2010 ACR/European League against Rheumatism (EULAR) classification criteria for RA [22] were included from the Department of Rheumatology of Sun Yat-sen Memorial Hospital. All patients had active disease, defined as a 28-joint disease activity score (DAS28) ≥2.6. Thirteen patients with osteoarthritis (OA) and ten with non-inflammatory orthopedic arthropathies (Orth.A), including five with meniscus injury, two with anterior cruciate ligament injuries, one with traumatic arthritis, one with medial plica syndrome, one with discoid meniscus, were included from the Department of Rheumatology and Orthopedics as less-inflamed disease controls [23]. Baseline demographic and clinical features of all patients are shown in Table 1. This study was conducted in accordance with the Declaration of Helsinki, and the protocol was approved by Medical Ethics Committee of Sun Yat-sen Memorial Hospital. All patients gave written informed consent.

**Table 1:** **Baseline demographic and clinical features of RA, OA and Orth.A patients in the study of synovial PGC-1β by immunohistochemistry**

**Characteristic**	**RA patients (n = 31)**	**OA patients (n = 13)**	**Orth.A patients (n = 10)**
**Demographic**			
Age, yrs, median (IQR)	61 (53 to 66)	61 (56 to 65)	32 (25 ~ 44)
Female, n (%)	24 (77.4)	11 (84.6)	5 (50.0)
**Disease status**			
Disease duration, mo, median (IQR)	36 (12 to 120)	60 (24 to 114)	3 (1 to 39)
ESR, mm/h, median (IQR)	82 (50 to 108)	18 (11 to 33)	14 (5 to 38)
CRP, mg/dl, median (IQR)	4.28 (1.89 to 6.05)	0.33 (0.25 to 0.93)	0.19 (0.08 to 0.34)
Rheumatoid factor-positive, n (%)	28 (90.3)	NA	NA
Anti-CCP-positive, n (%)	26 (83.9)	NA	NA
DAS28, median (IQR)	5.78 (4.83 to 6.39)	NA	NA
**Previous medications, n (%)**		NA	NA
Corticosteroids	12 (38.7)	NA	NA
Methotrexate	12 (38.7)	NA	NA
Leflunomide	5 (16.1)	NA	NA
Sulfasalazine	1 (3.2)	NA	NA
Hydroxychloroquine	3 (9.7)	NA	NA
Etanercept	3 (9.7)	NA	NA

ESR, erythrocyte sedimentation rate; CRP, C-reactive protein; RF, rheumatoid factor; Anti-CCP, anti-cyclic citrullinated peptide antibodies; DAS28, disease activity score 28-joint assessment; n, number of patients; RA, rheumatoid arthritis; OA, osteoarthritis; Orth.A, orthopedic arthropathies; NA, not applicable.

### Clinical assessments

Clinical data of all patients with RA were collected at baseline, including the 28-joint tender and swollen joint count (28TJC and 28SJC), patient and provider global assessment of disease activity (PtGA and PrGA), pain visual analog scale (pain VAS), Chinese language version of Stanford health assessment questionnaire (HAQ) [24], erythrocyte sedimentation rate (ESR), C-reactive protein (CRP); rheumatoid factor (RF), and anti-cyclic citrullinated peptide antibody (anti-CCP). Disease activity was assessed with DAS28 with four variables, including CRP (DAS28 (4)-CRP) [25].

### Synovial tissue collection and FLS culture

RA synovium was collected by closed needle biopsy [26]. At least six pieces of synovial tissue were obtained per patient to minimize sampling error [27]. The OA and Orth.A specimens were obtained from knees by closed needle biopsy, arthroplasty or arthroscopy. All samples were fixed in 10% neutral formalin and embedded in paraffin. Sections (5 μm) were cut serially and mounted on adhesive glass slides. Sealed slides were stored at –20°C until staining.

FLS were isolated from the synovial tissues by modified tissue culture method [28]. Fresh synovial tissues were minced and digested in type I collagenase (Sigma-Aldrich, St Louis, MO, USA). The cells were cultured with DMEM-Ham’s F-12 (DMEM/F12) (Gibco, Life Technologies, Shanghai, China), containing 20% fetal calf serum (Gibco, Life Technologies, Mulgrave, VIC, Australia) in a humidified 5% CO_2_ incubator. FLS from passages three to five were used in this study.

### Immunohistochemistry

Serial sections of synovial tissues were stained with H&E and a three-step immunoperoxidase method for immunohistochemistry. Sections were incubated with the PGC-1β (Bioss, Beijing, China) at 1/200 dilution overnight at 4°C after deparaffinization and retrieval. The sections were incubated with EnVision Mouse or Rabbit conjugate (Dako, Carpinteria, USA) for 30 minutes at 37°C. The 3,3-diaminobenzidine (DAB)-positive substrate was used for the color reaction. Sections were counterstained with hematoxylin. Nonspecific isotype IgG was used as a negative control. The percentage of PGC-1β-positive-staining cells in the lining layers and sublining area were determined by manual observation of five different fields at magnification × 400, respectively.

### Immunofluorescence

FLS were incubated with primary antibody recognizing PGC-1β (Bioss, Beijing, China) or normal rabbit IgG (control) after fixation, permeablization and blocking. FLS were stained with Alexa Fluor 488-conjugated goat anti-mouse IgG diluted 1:1,000 (Invitrogen,  Carlsbad, USA), and co-stained with 4′6-diamindino-2-phenylindole (DAPI) (Sigma-Aldrich, St Louis, MO, USA) to visualize nuclei. FLS were analyzed by confocal microscopy (Zeiss, Jena, Germany).

### Lentivirus infection in RA-FLS

Gene knockdown was performed using lentivirus sh-RNA, which was synthesized by Shanghai GeneChem Co Ltd (Shanghai, China). Short hairpin RNA (shRNA) were cloned into pLKO.1 (GV248) lentiviral vectors. The sh-PGC-1β targeting sequence was CAGATACACTGACTACGAT. Culture supernatants containing sh-RNA were added to RA-FLS in the presence of polybrene. The cells were selected using 1 μg/ml puromycin after 24 h. Stable cell lines were verified by western blot.

### Plasmid construction and transfection

The fragment of human PGC-1β was amplified from the cDNA of 293T cells by using specific primers (forward primer 5′-TTCAAGCTTATGGCGGGGAACGACTG-3′ and reverse primer 5′-ATCTCGAGTCAATGCAGGCTCTGCTG-3′) with the HindIII and XhoI restriction sites. Following the conditions at 35 cycles at 94°C for 1 minute, 58°C for 180 s, and 72°C for 1 minute, their action was continued for 10 minutes at 72°C after the last cycle using Ex Taq Polymerase (Takara, Otsu, Japan). The PCR product was purified and digested with HindIIIand Xho, then cloned into a mammalian expression vector pcDNA3.1 (+) with the corresponding restriction sites (Novagen, Darmstad, Germany). The recombinant plasmid was confirmed by DNA sequencing. RA-FLS were cultured in 6-well cell culture plates to 80 to 90% confluency and then subjected to transfection using the lipofectamine TM 2000 reagent (Life Technologies, Carlsbad, USA) according to the manufacturer’s instructions. Cells were harvested 72 h after transfection.

### Apoptosis detection assay and cell cycle analysis

The apoptosis of stably transduced RA-FLS was measured through detecting APC-conjugated Annexin-V and 7-AAD by flow cytometry (FCM) (BD PharMingen, San Diego, USA). For cell cycle analysis, they were fixed with cold ethanol overnight and then treated with propidium iodide and RNase before fluorescence-activated cell sorting (FACS) analysis. The cell cycle distribution was determined using flow cytometry (FCM). Apoptosis and cell cycle were quantified using FACS and CellQuest software (BD Biosciences, Mountain View, USA).

### Quantitative real-time PCR (qPCR)

Total RNA was prepared from cells using RNAiso Reagent (Takara, Otsu, Japan). Complementary DNA (cDNA) samples were synthesized with reverse-transcription kit (Takara, Otsu, Japan). Amplification of the cDNA was performed using specific oligonucleotide primers (Table 2). The qPCR was performed using QuantiTeckTM SYBR Green PCR kit (Takara, Otsu, Japan). The reactions were initiated with denaturation of cDNA templates at 95°C for 30 s, 95°C for 5 s and 60°C for 30 s and amplification for 40 to 50 cycles. Samples were run in triplicate in a Roche LightCycler480 sequence detector system (Roche, Basel, Switzerland).

**Table 2:** **Primers for quantitative real-time PCR**

**Name**	**Sense primer (5′-3′)**	**Antisense primer (5′-3′)**
β-actin	GGACTTCGAGCAAGAGATGG	TGTGTTGGCGTACAGGTCTTTG
IL-6	CTGCGCAGCTTTAAGGAGTTC	CAATCTGAGGTGCCCATGCTA
IL-8	GTGCAGAGGGTTGTGGAGAAGTTT	TCACTGGCATCTTCACTGATTCTTG
IL-1β	CCAGCTACGAATCTCCGACC	CATGGCCACAACAACTGACG
TNF-α	GCTAAGAGGGAGAGAAGCAACTACA	GAAGAGGCTGAGGAACAAGCA
MMP-3	TTTCCAGGGATTGACTCAAAGA	AAGTGCCCATATTGTGCCTTC
MMP-13	TCCTGGGCCAAATTATGGAG	TTGCCGGTGTAGGTGTAGATAGGAA
RANKL	ACCAGCATCAAAATCCCAAG	CCCCAAAGTATGTTGCATCC
NF-κB	ATGTGGAGATCATTGAGCAGC	CCTGGTCCTGTGTAGCCATT
ERK	CTACACGCAGTTGCAGTACAT	CAGCAGGATCTGGATCTCCC
p38	CCCGAGCGTTACCAGAACC	TCGCATGAATGATGGACTGAAAT

MMP: matrix metalloproteinase; RANKL, receptor activator of nuclear factor-kappa B ligand; ERK, extracellular signal-regulated kinase.

### Western blot analysis

Protein lysates from cells were subjected to SDS-PAGE and target proteins were detected with primary antibodies recognizing p-p38, p38, P-extracellular signal-regulated kinase (P-ERK), ERK, p-JNK, JNK, p-NF-κB p65 (Ser536), NF-κβ p65 and glyceraldehyde-3-phosphate dehydrogenase (GAPDH) (CST, Danvers, Massachusetts, USA), PGC-1β and RANKL (Epitomics, San Francisco, USA), MMP-3 and MMP-13 (Abcam, Cambridge, USA) respectively. After incubation with appropriate horseradish peroxidase (HRP)-conjugated secondary antibodies (EarthOx, Millbrae, California, USA), protein bands were visualized using enhanced chemiluminescence (Millipore, Boston, USA) plus western blot detection reagents followed by exposure to a scanning imager (G:BOX Gel & Blot Imaging Series from Syngene, Cambridge, UK).

### Determination of proinflammatory cytokine levels

Proinflammatory cytokines including IL-8, IL-6, IL-1β, and TNF-α in the culture supernatants were quantified using cytometric bead array (CBA) human inflammatory cytokines kit (BD Biosciences, San Jose, USA). The cytokines were analyzed by standard curves plotted using a four-parameter logistic curve-fitting model.

### Dual-luciferase reporter gene assays

Luciferase reporter construct containing the NF-κB promoter region (wild type, WT) or a construct with mutated sites (MUT) was cloned into pGL3-based vectors, then temporarily transfected with 1 μg of the promoter reporter plasmid into RA-FLS using the Lipofectamine 2000 (Invitrogen, Shanghai, China), while 40 ng of the phRL-TK plasmid were co-transfected into the cells to verify transfection efficiency. Then, RA-FLS cells were co-transfected with pcDNA3.1-PGC-1β to increase PGC-1β expression, and empty vector pcDNA3.1 to keep total DNA concentration constant. After 36 h of transfection, the luciferase activities were measured on a spectraMax M5 reader (Molecular Devices, California, USA) using the Dual-Luciferase Reporter Assay System (Promega, Madison, USA).

### Statistical analysis

Statistical analyses were performed with SPSS 13.0 statistical software (SPSS Inc., Chicago, IL, USA). For categorical variables, data were presented as frequencies and percentages. For continuous variables, data were presented as mean and SD, or median and IQR. Nonparametric tests (Mann-Whitney rank-sum test between two groups, Kruskal-Wallis one-way analysis of variance on ranks among three or more groups for continuous variables) were used to compare the differences in PGC-1β expression in synovium and FLS. Spearman’s rank order correlation test was used for assessing the correlation between PGC1-1β expression in RA-FLS and both clinical indicators and proinflammatory cytokines. Student’s *t*-test was used to assess the differences in proinflammatory cytokines, MMPs and RANKL between the experimental and control groups. Two tailed *P*-values <0.05 were considered statistically significant.

## Results

### Characteristics of the study patients

Baseline demographic and clinical features of all patients are shown in Table 1. Age and sex did not differ among the patients with RA and OA. Among the patients with RA, 45% (14/31) had never taken corticosteroids or disease-modifying anti-rheumatic drugs (DMARDs). These patients had taken only Chinese herbal medicine and/or painkillers to relieve arthralgia. At recruitment, 13% (4/31) had taken corticosteroids alone. The remaining 42% (13/31) received treatment with one or more DMARDs, including methotrexate, leflunomide, sulfasalazine, hydroxychloroquine, or etanercept.

### Expression of PGC-1β is upregulated in synovium in RA

**Figure 1:**
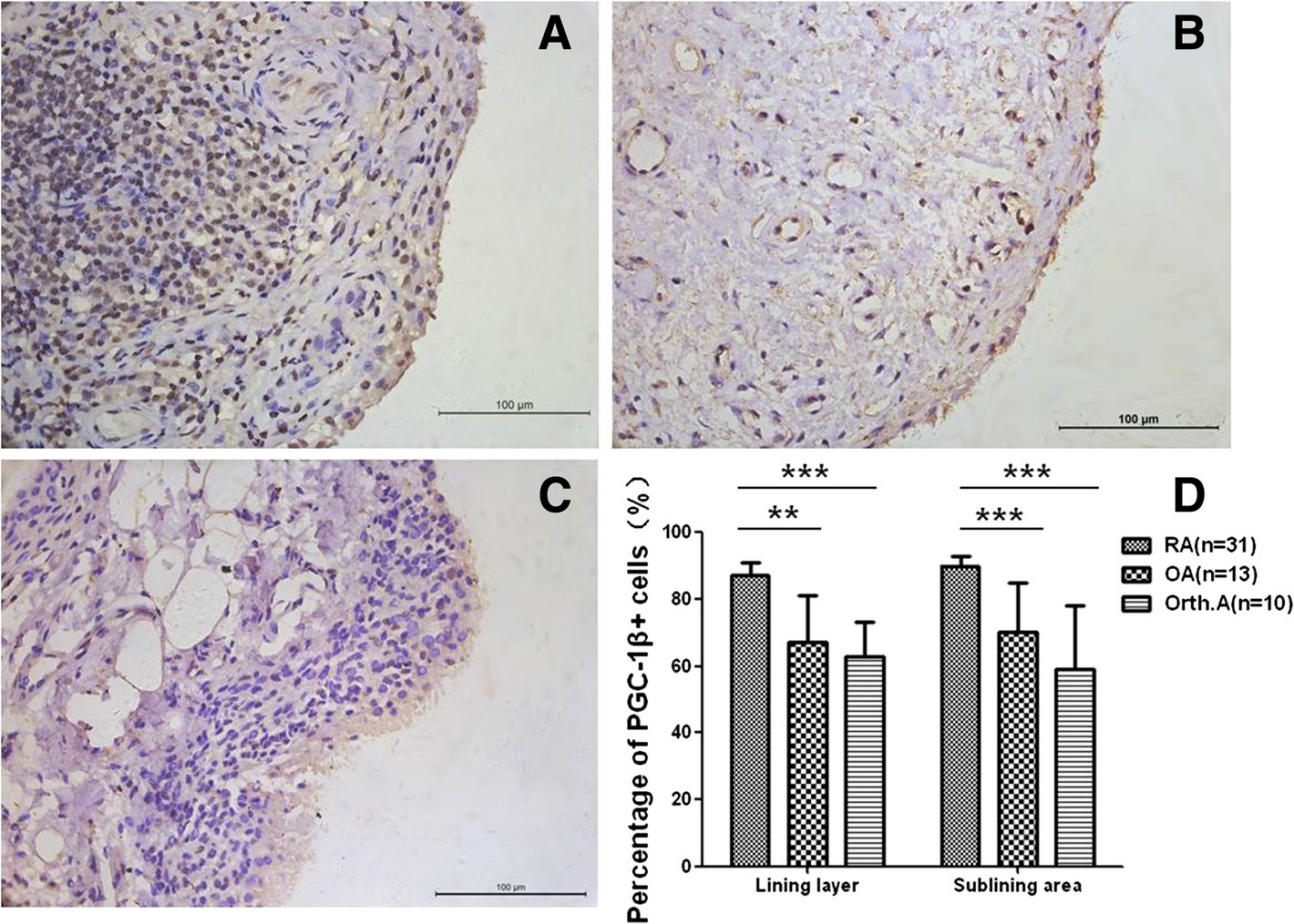
**Expression of peroxisome proliferator-activated receptor-gamma coactivator-1 β (PGC-1β) is upregulated in synovium in rheumatoid arthritis (RA). (A)** Intensive synovial PGC-1β expression in synovium of a RA patient. **(B)** Moderate synovial PGC-1β expression in synovium of an osteoarthritis (OA) patient. **(C)** Mild synovial PGC-1β expression in synovium of an orthopedic arthropathy (Orth.A) patient. **(D)** Percentage of lining PGC-1β + cells in RA, OA and Orth.A patients. **A**, **B**, **C**: original magnification × 400. The data are represented by median ± IQR. **P <0.01, ***P <0.001.

PGC-1β expression in RA synovium was observed with intense nuclear staining in lining cells (both macrophage-like synoviocytes and fibroblast-like synoviocytes) and sublining inflammatory cells (mostly in lymphocytes and plasma cells). As shown in Figure 1, the percentage of PGC-1β + lining cells (median 87%, IQR 78% to 91%) was significantly higher than that in OA (median 69%, IQR 47% to 81%) or in Orth.A (median 63%, IQR 48% to 69%). In the sublining area, the percentage of PGC-1β + lining cells (median 90%, IQR 86% to 93%) was significantly higher than that in OA (median 71%, IQR 60% to 86%) or in Orth.A (median 60%, IQR 50% to 78%).

### PGC-1β is over-expressed in RA-FLS

**Figure 2:**
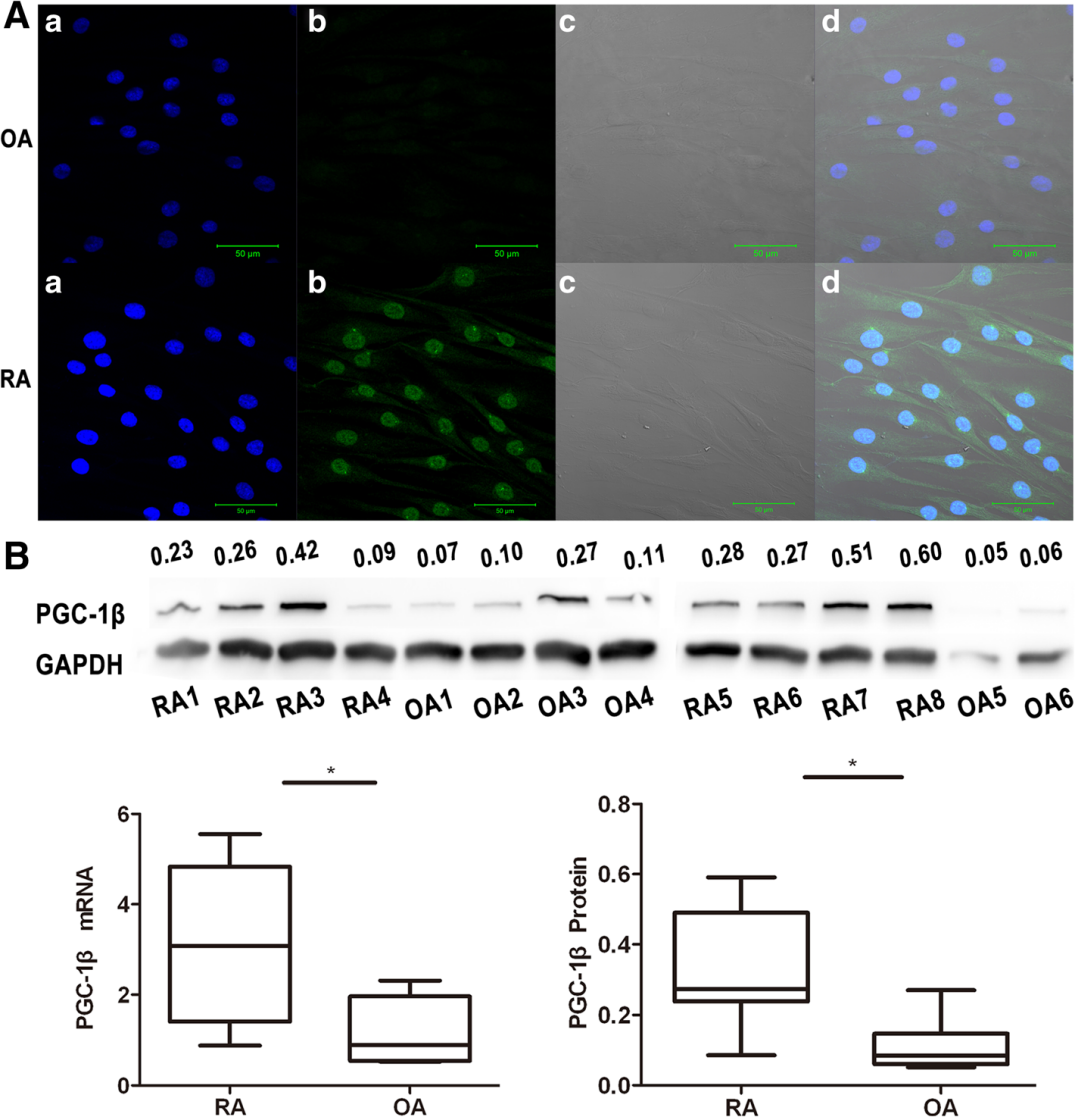
**Expression of peroxisome proliferator-activated receptor-gamma coactivator-1 β (PGC-1β) is over-expressed in rheumatoid arthritis (RA)-fibrolast-like synoviocytes (FLS). (A)** Immunofluorescence staining of PGC-1β in primary cultures of FLS from osteoarthritis (OA) and RA patients. (a, DAPI (blue); b, PGC-1β (green); c, neutral light; d, merged a, b with c. a, b, c: original magnification × 400). **(B)** Left panel: PGC-1β mRNA expression in FLS from RA (n = 8) compared with that from OA (n =6) evaluated by qPCR. Right panel: PGC-1β protein level in FLS from RA patients (n = 8) and OA controls (n = 6) was detected by western blot. The intensity for each band was densitometrically quantified and normalized against the intensity of glyceraldehyde-3-phosphate dehydrogenase (GAPDH). The data are represented by mean ± SD. *P <0.05.

The expression of PGC-1β mRNA and protein in RA-FLS was detected from eight RA patients and six OA patients. Their baseline demographic and clinical features are shown in Table 3. Immunofluorescence staining showed higher expression of PGC-1β in RA-FLS compared with OA-FLS (Figure 2A). The expression of PGC-1β mRNA and protein in RA-FLS was significantly enhanced compared with OA controls (3.18 ± 1.72 versus 1.17 ± 0.74, *P* = 0.019; 0.33 ± 0.17 versus 0.11 ± 0.08, *P* = 0.014, respectively) (Figure 2B).

**Table 3:** **Baseline demographic and clinical features of RA and OA patients in the study of PGC-1β expression in FLS by qPCR and western blot**

**Characteristic**	**RA patients (n = 8)**	**OA patients (n = 6)**
**Demographic**		
Age, yrs, median (range)	55 (33 to 70)	57 (51 to 68)
Female, n (%)	8 (100)	6 (100)
**Disease status**		
Disease duration, mo, median (range)	78 (2 to 240)	36 (12 to 168)
ESR, mm/h, median (range)	50 (11 to 83)	18 (12 to 32)
CRP, mg/dl, median (range)	1.53 (0.02 to 4.55)	0.33 (0.08 to 1.11)
Rheumatoid factor-positive, n (%)	7 (87.5)	NA
Anti-CCP-positive, n (%)	8 (100)	NA
DAS28, median (range)	4.36 (3.06 to 5.82)	NA
**Previous medications, n (%)**		NA
Corticosteroids	3 (37.5)	NA
Methotrexate	4 (50)	NA
Leflunomide	3 (37.5)	NA
Hydroxychloroquine	2 (25)	NA
Infliximab	1 (12.5)	NA
Etanercept	1 (12.5)	NA

FLS, fibrolast-like synoviocytes; ESR, erythrocyte sedimentation rate; CRP, C-reactive protein; RF, rheumatoid factor; Anti-CCP, anti-cyclic citrullinated peptide antibodies; DAS28, disease activity score 28-joint assessment; n, number of patients; RA, rheumatoid arthritis; OA, osteoarthritis; NA, not applicable.

### RA-FLS PGC-1β protein expression shows positive correlation with clinical parameters

Moreover, significant correlations were found between FLS PGC-1β expression and CRP, ESR or DAS28 from eight RA patients (CRP: *r* = 0.738, *P* = 0.037; ESR: *r* = 0.762, *P* = 0.028; DAS28: *r* = 0.786, *P* = 0.021). There was no significant correlation between PGC-1β expression and RF, anti-CCP, 28TJC, 28SJC, PtGA, PrGA, pain VAS or HAQ (all *P* >0.05).

### PGC-1β knockdown attenuates proinflammatory cytokines, MMPs and RANKL production in RA-FLS

**Figure 3:**
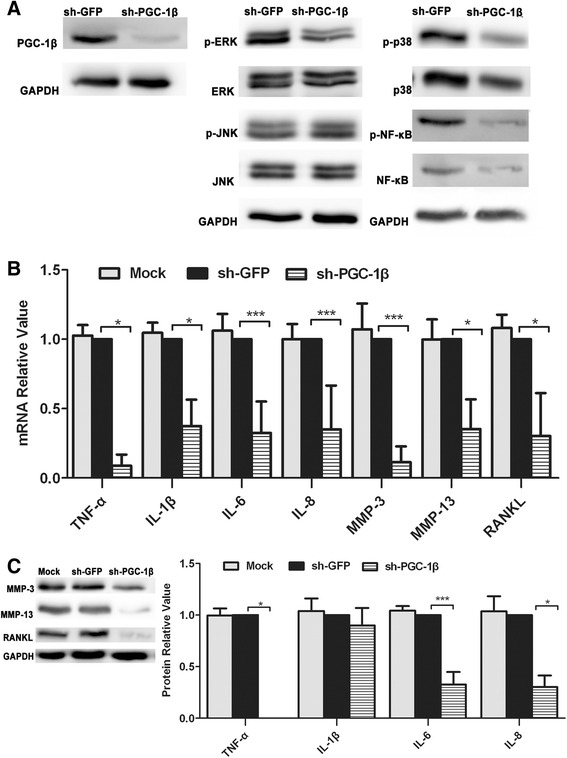
**Peroxisome proliferator-activated receptor-gamma coactivator-1 β (PGC-1β) knockdown attenuates proinflammatory cytokines, matrix metalloproteinases (MMPs) and receptor activator of nuclear factor-kappa B ligand (RANKL) production in rheumatoid arthritis (RA)-fibrolast-like synoviocytes (FLS). (A)** After PGC-1β knockdown, the protein level of mitogen-activated protein kinases (MAPKs) in FLS was detected by western blot. **(B)** After PGC-1β knockdown, the proinflammatory cytokines, MMPs and RANKL mRNA expression in FLS was evaluated by qPCR. **(C)** After PGC-1β knockdown, the protein level of proinflammatory cytokines was examined by cytometric bead array, while the level of MMP-3, MMP-13 and RANKL was detected by western blot. The data are represented by mean ± SD from three independent experiments. *P <0.05, **P <0.01, ***P <0.001.

To determine the role of PGC-1β in RA-FLS proinflammatory cytokine production, the proinflammation production in PGC-1β knockdown RA-FLS was compared with negative control (NC) infected with sh-GFP. The protein expression of PGC-1β in RA-FLS transfected with sh-PGC-1β was hardly detected compared with NC (Figure 3A). PGC-1β knockdown suppressed the expression of TNF-α, IL-6, IL-8, RANKL, MMP-3 and MMP-13 relative to the NC, but not IL-1β (Figure 3B and 3C). To explore whether this effect is mediated through inhibition of the MAPK and NF-κB signaling pathway, MAPK and NF-κB expression were evaluated. From these results, decreased expression of PGC-1β visibly suppressed the activity of ERK, p38 and NF-κB, but not JNK (Figure 3A).

### PGC-1β overexpression enhances proinflammatory cytokines, MMPs and RANKL production in RA-FLS

**Figure 4:**
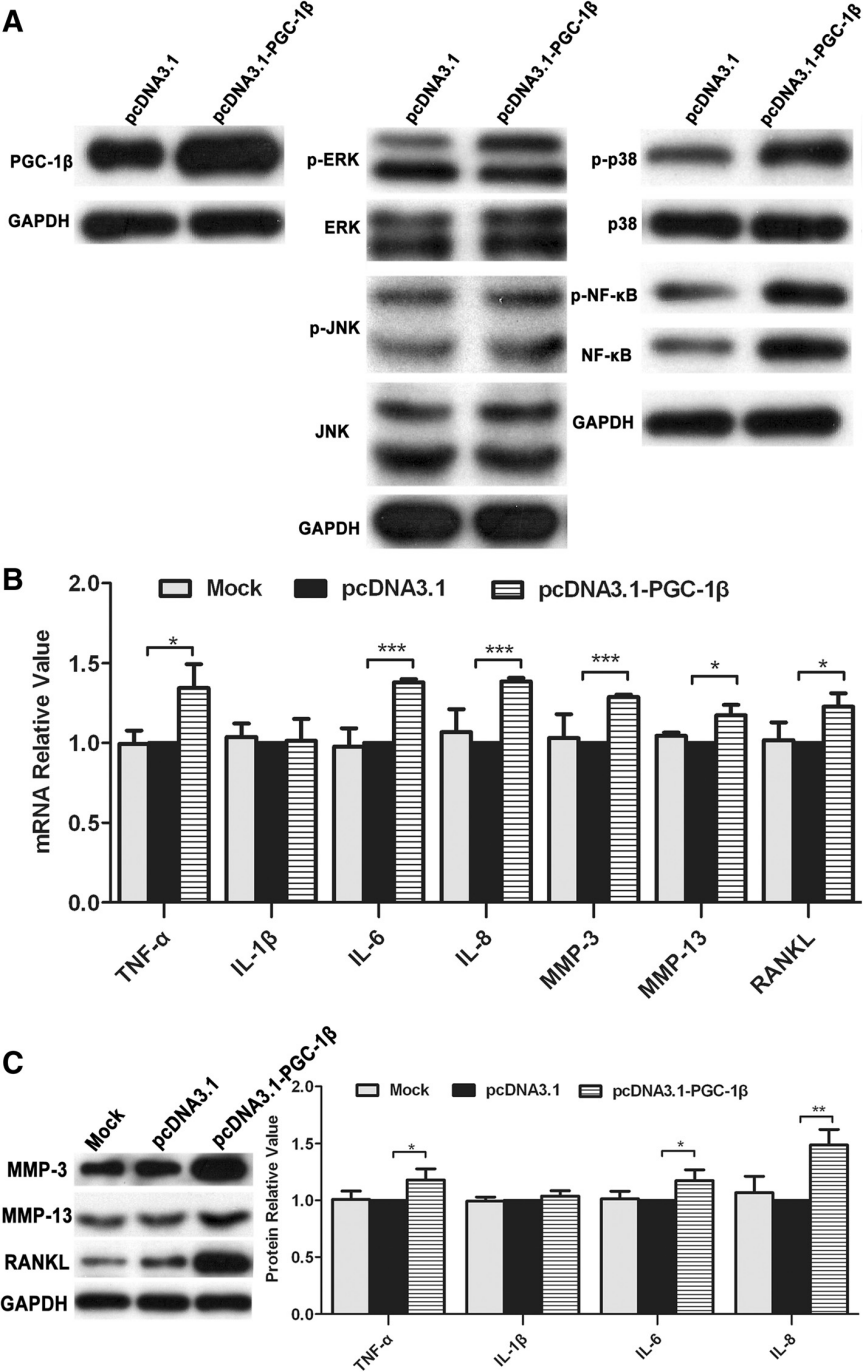
**Peroxisome proliferator-activated receptor-gamma coactivator-1 β (PGC-1β) overexpression enhances proinflammatory cytokines, matrix metalloproteinases (MMPs) and receptor activator of nuclear factor-kappa B ligand (RANKL) production in rheumatoid arthritis (RA)-fibrolast-like synoviocytes (FLS). (A)** After PGC-1β overexpression, the protein level of mitogen-activated protein kinases (MAPKs) in FLS was detected by western blot. **(B)** After PGC-1β overexpression, the proinflammatory cytokines, MMPs and RANKL mRNA expression in FLS was evaluated by qPCR. **(C)** After PGC-1β overexpression, the protein level of proinflammatory cytokines was examined by cytometric bead array, while the level of MMP-3, MMP-13 and RANKL was detected by western blot. The data are represented by mean ± SD from three independent experiments. *P <0.05, **P <0.01, ***P <0.001.

To determine whether PGC-1β overexpression enhanced proinflammatory cytokines production, RA-FLS were transfected with pcDNA3.1-PGC-1β or pcDNA3.1control. As shown in Figure 4A, PGC-1β protein expression was significantly overexpressed in RA-FLS transfected with pcDNA3.1-PGC-1β compared with cells transfected with pcDNA3.1 control. PGC-1β overexpression remarkably exacerbated RA-FLS-mediated inflammation, as evidenced by upregulating TNF-α, IL-6, IL-8, RANKL, MMP-3 and MMP-13 expression, but not IL-1β (Figure 4B and 4C). A detailed analysis of downstream signaling pathways was performed to detect the levels of phosphorylated MAPKs and NF-κB. The levels of ERK, p38 and NF-κB phosphorylation increased after PGC-1β overexpression in RA-FLS (Figure 4A).

### PGC-1β knockdown enhances apoptosis in RA-FLS

**Figure 5:**
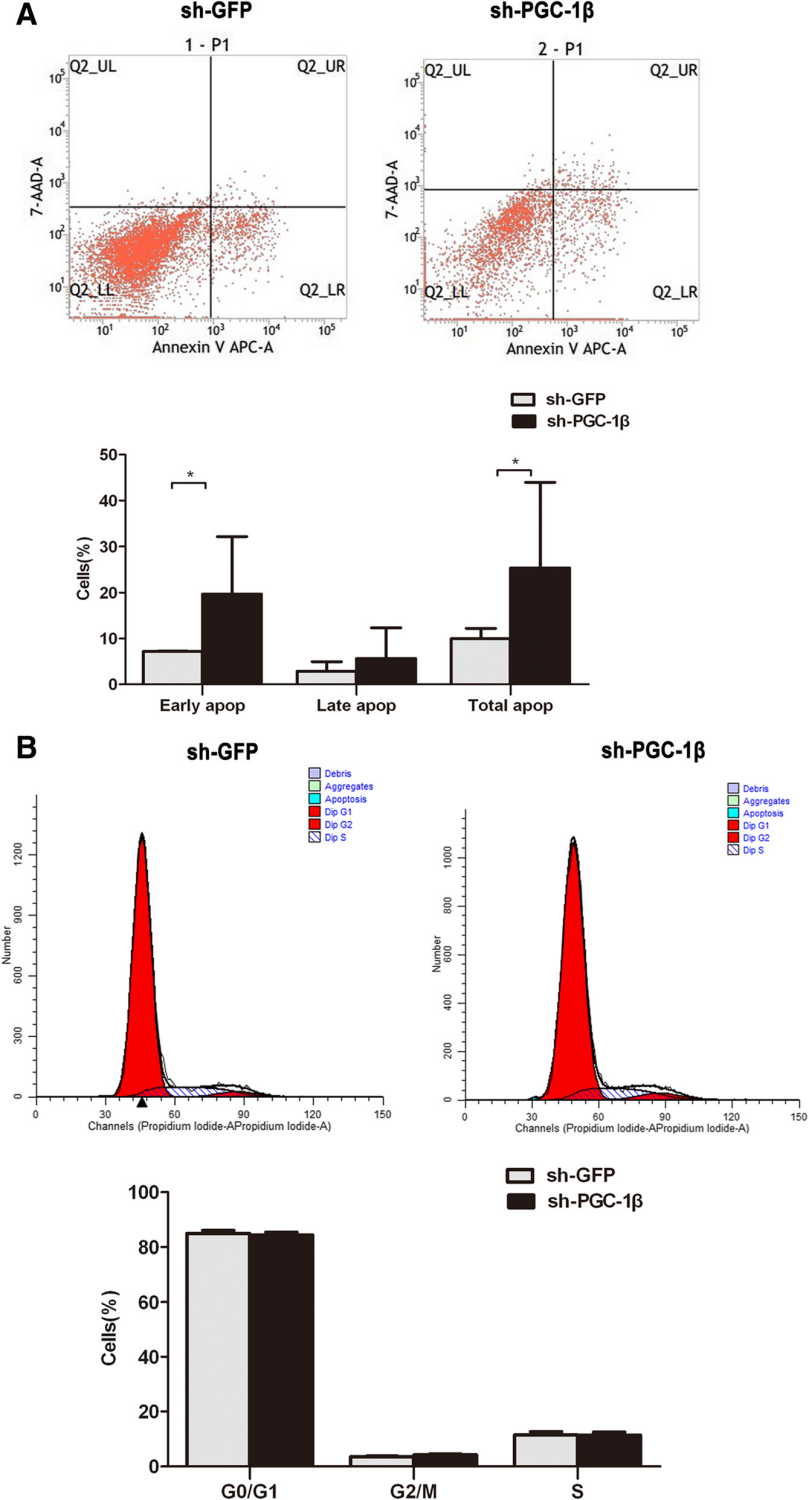
**Peroxisome proliferator-activated receptor-gamma coactivator-1 β (PGC-1β) knockdown enhances apoptosis in rheumatoid arthritis (RA)-fibrolast-like synoviocytes (FLS). (A)**. Flow cytometric analysis demonstrating the effect of PGC-1β knockdown on cell apoptosis. **(B)** Flow cytometric analysis demonstrating the effect of PGC-1β knockdown on cell cycle progression. The data are represented by mean ± SD from three independent experiments. *P <0.05.

The results of Annexin V APC-A and PI FCM analyses showed that lentivirus-mediated inhibition of PGC-1β significantly increased early apoptosis and total apoptosis except late apoptosis relative to the NC (early apoptosis: 7.15 ± 0.10 versus 20 ± 12.11, *P* = 0.026; late apoptosis: 2.85 ± 2.10 versus 5.64 ± 6.69, *P* = 0.110; total apoptosis: 10 ± 2.20 versus 25.30 ± 18.67, *P* = 0.026) in RA-FLS (Figure 5A). To assess whether this effect was mediated through perturbation of the cell cycle, cellular subpopulations at various phases of the cell cycle were analyzed. The results showed no significant change at different phases relative to the NC (Figure 5B).

### PGC-1β promotes NF-κB transcription in RA-FLS

**Figure 6:**
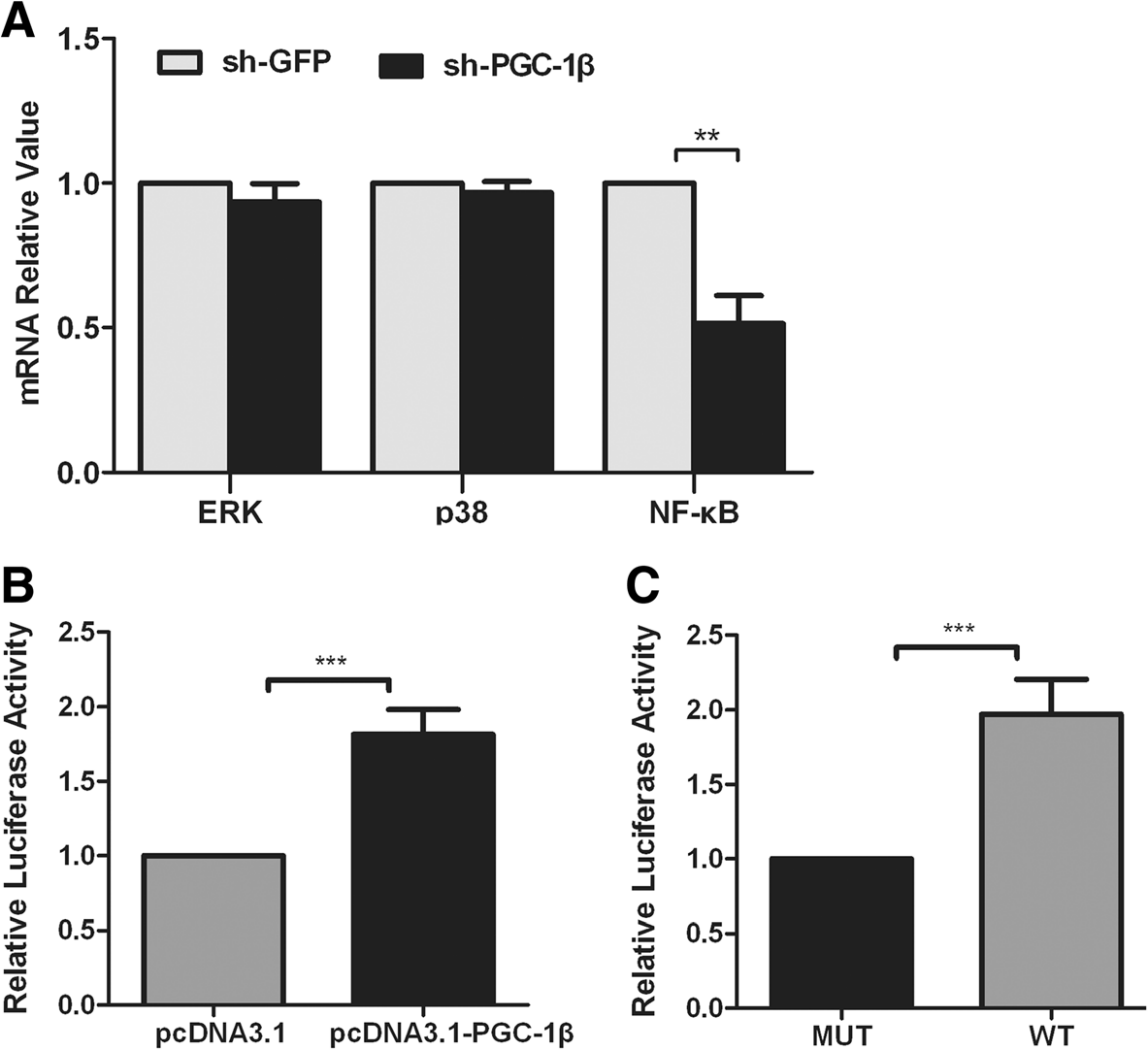
**Peroxisome proliferator-activated receptor-gamma coactivator-1 β (PGC-1β) promotes NF-κB transcription in rheumatoid arthritis (RA)-fibrolast-like synoviocytes (FLS). (A)**. After PGC-1β overexpression, the mRNA expression of ERK, p38 and NF-κB in FLS was evaluated by qPCR. **(B)** After RA-FLS transfected with a wild type-NF-κB reporter region (WT) and plasmid pcDNA3.1-PGC-1β or pcDNA3.1, the luciferase activities were measured on a spectraMax M5 reader. **(C)** After RA-FLS transfected with plasmid pcDNA3.1-PGC-1β and WT region or a mutated-NF-κB reporter construct (MUT), the luciferase activities were measured on a spectraMax M5 reader. The data are represented by mean ± SD from three independent experiments. **P <0.01, ***P <0.001.

To assess whether PGC-1β indirectly or directly mediates ERK, p38 and NF-κB transcription, the mRNA expression of ERK, p38 and NF-κB in PGC-1β knockdown RA-FLS were compared with NC infected with sh-GFP. Knockdown of PGC-1β significantly suppressed mRNA expression of NF-κB except ERK and p38 (Figure 6A), which suggests that PGC-1β may directly mediate NF-κB transcription.

To experimentally validate whether PGC-1β mediated NF-κB transcription, reporter gene assays were performed. Co-transfection of pcDNA3.1-PGC-1β resulted in an increase in NF-κB luciferase activity compared with co-transfection of pcDNA3.1 (Figure 6B). PGC-1β progressively increased NF-κB luciferase activity in WT more than in MUT (Figure 6C). These results suggest that PGC-1β directly promoted NF-κB transcription.

## Discussion

RA is an autoimmune systemic inflammatory disease that causes progressive synovial inflammation resulting in irreversible joint destruction, chronic disability and premature mortality [10]. RA-FLS are key mediators of cartilage and bone destruction. Activated RA-FLS play crucial roles in determining the site where inflammation occurs and in the subsequent maintenance of persistent inflammation in the joint microenvironment [29]. They act directly on cartilage through secretion of MMPs, cathepsins and inflammatory cytokines, and indirectly on bone by regulating osteoclastogenesis [30,31]. Therefore, inhibition of proinflammatory cytokine, MMP and RANKL production in RA-FLS constitutes an important target for novel therapeutic approaches that hinder the destruction of cartilage and bone [32]. It has been demonstrated that NF-κB and MAPK are central regulators in inflammatory processes, including the pathophysiological development of RA [33,34]. Activation of NF-κB and MAPKs robustly induce proinflammatory cytokines, MMPs and RANKL production in RA-FLS [32,35].

PGC-1β is a transcriptional coactivator that regulates diverse signal transduction pathways [16], and has been reported to exert protective effects through inhibition of NF-κB activity [17]. PGC-1α overexpression vigorously has been shown to inhibit p38 MAPK phosphorylation [19]. It remains unknown as to whether PGC-1β plays a role in the regulation of RA-FLS inflammation. To the best of our knowledge, this is the first study investigating the effects of PGC-1β on inflammation in RA. In the present study, we found that PGC-1β was expressed in lining cells (both macrophage-like synoviocytes and fibroblast-like synoviocytes) and sublining inflammatory cells (mostly in lymphocytes and plasma cells), and PGC-1β expression in RA synovium was significantly higher than that in OA or Orth.A synovium. Consistently, PGC-1β expression was more notably upregulated in RA-FLS than in OA-FLS. We also observed significant positive correlation of RA-FLS PGC-1β expression with clinical parameters of disease activity, including DAS28, CRP and ESR. Thus, we postulate that elevated PGC-1β expression may be potentially involved in the pathogenesis of inflammation in RA.

To further explore the effect of PGC-1β on RA-FLS-mediated inflammation, we used lentivirus sh-RNA to inhibit PGC-1β expression and pcDNA3.1-PGC-1β to enhance PGC-1β expression. The results showed that the expression of TNF-α, IL-6, IL-8, MMP-3, MMP-13 and RANKL, and the activity of ERK, p38 and NF-κB, were clearly restrained in PGC-1β knockdown RA-FLS. Conversely, the expression of TNF-α, IL-6, IL-8, MMP-3, MMP-13 and RANKL, and the activity of ERK, p38 and NF-κB were visibly increased in PGC-1β overexpression RA-FLS. Several lines of evidence have demonstrated that inhibition of ERK, p38 and NF-κB activation dampened TNF-α, IL-6, IL-8, MMP-3, MMP-13 and RNAKL expression in RA-FLS [35-38]. We therefore hypothesized that PGC-1β mediated the expression of TNF-α, IL-6, IL-8, MMP-3, MMP-13 and RANKL through ERK, p38 and NF-κB. This is further substantiated by a study that TNF-α expression was lower in muscle-specific PGC-1α knockout mice, and TNF-α expression increased in muscle-specific PGC-1α overexpression mice compared with littermate WT mice [39]. Contrary to that study, it was reported that PGC-1β overexpression resulted in the attenuation of macrophage-mediated inflammation by strongly inhibiting proinflammatory cytokine production [20]. The role of PGC-1β appears very diverse and the proinflammatory or anti-inflammatory effects of PGC-1β in different disease models require further study.

In our study, we found that PGC-1β mediated proinflammatory cytokine production without IL-1β, but the specific mechanism remains unclear. Recently, Promsong *et al*. reported that ellagic acid resulted in an increase of IL-1β and a decrease of IL-6, IL-8 and TNF-α in oral epithelial cells [40]. In addition, the wild *Legionella pneumophila* strain has been shown to increase the levels of IL-8, IL-6, and TNF-α but not IL-1β in human pulmonary carcinoma cells [41]. Therefore, self-regulation of PGC-1β-mediated IL-6, IL-8 and TNF-α expression, but not IL-1β, might be attributed to the difference in existing pathways regulating IL-1β, IL-6, IL-8 and TNF-α production.

It has been established that defective apoptosis contributes to synovial hyperplasia in RA. Promoting RA-FLS apoptosis is a new treatment strategy for RA [42]. In the present study, we found increased early apoptosis and total apoptosis in PGC-1β knockdown RA-FLS. However, the cell cycle showed no significant change after PGC-1β suppression, which may imply that PGC-1β affects RA-FLS apoptosis through a molecular mechanism other than the cell cycle. Previous research has indicated that ERK, p38 and NF-κB negatively regulates RA-FLS apoptosis [43-46]. We postulated that suppression of PGC-1β might promote RA-FLS apoptosis through inhibition of ERK, p38 and NF-κB activation.

Our results also showed that PGC-1β downregulation significantly inhibited the mRNA expression of NF-κB, except for ERK and p38, which indicates that PGC-1β might mediate NF-κB transcription. To further validate PGC-1β-mediated NF-κB transcription, we performed reporter gene assays. The assays showed that PGC-1β overexpression resulted in increased NF-κB luciferase activity, which indicated that PGC-1β directly promoted NF-κB transcription. The result was consistent with a previous study showing that PGC-1 downregulation decreases NF-κB transcription [47]. The precise mechanism for the interaction between PGC-1β and ERK or p38 remains unclear and requires further study.

## Conclusions

The results of this study show that suppression of PGC-1β attenuates the expression of TNF-α, IL-6, IL-8, MMP-3, MMP-13 and RANKL, and enhances apoptosis through inhibition of p38, ERK and NF-κB activation in RA-FLS, which suggests that blocking PGC-1β expression may provide a novel target for the treatment of RA.

## Abbreviations

28SJC: 28-joint swollen-joint count
28TJC: 28-joint tender-joint count
anti-CCP: anti-cyclic citrullinated peptide antibody
CBA: cytometric bead array
CRP: C-reactive protein
DAPI: 4’6-diamindino-2-phenylindole
DAS28: disease activity score 28-joint assessment
DAS28 (4)-CRP: 28-joint disease activity score with four variables, including C-reactive protein
DMARD: disease-modifying anti-rheumatic drug
DMEM/F12: Dulbecco’s modified Eagle’s medium (DMEM)-Ham’s F-12
ERK: extracellular signal-regulated kinase
ESR: erythrocyte sedimentation rate
FACS: fluorescence-activated cell sorting
FCM: flow cytometry
FLS: fibrolast-like synoviocytes
GAPDH: glyceraldehyde-3-phosphate dehydrogenase
H&E: hematoxylin and eosin
HAQ: Chinese language version of Stanford Health Assessment Questionnaire
IL: interleukin
IQR: interquartile range
JNK: c-Jun N-terminal kinase
MAPK: mitogen-activated protein kinase
MMP: matrix metalloproteinase
MUT: mutated sites
NC: negative control
NF-κB: nuclear factor-kappaB
OA: osteoarthritis
Orth.A: orthopedic arthropathies
p38: p38 mitogen-activated protein kinase
pain VAS: pain visual analog scale
PGC-1β: peroxisome proliferator-activated receptor-gamma coactivator-1 beta
PrGA: provider global assessment of disease activity
PtGA: patient global assessment of disease activity
qPCR: quantitative real-time polymerase chain reaction
RA: rheumatoid arthritis
RANKL: receptor activator of nuclear factor-kappa B ligand
RF: rheumatoid factor
shRNA: short hairpin RNA
TNF-α: tumor necrosis factor alpha
WT: wild type
